# Supplementary material for: COZOID: contact zone identifier for visual analysis of protein-protein interactions
Source: BMC Bioinformatics. 2018 Apr 6;19:125. doi: 10.1186/s12859-018-2113-6 (PMC5889581; doi:10.1186/s12859-018-2113-6)
Supplement: Supplementary file 2 — Software build. Executable binary file of the software tool. (ZIP 118,784 kb) [file 12859_2018_2113_MOESM2_ESM.zip › cozoid/java/docs/beansbinding-1.2.1-doc.zip!/org/jdesktop/el/ResourceBundleELResolver.html]

ResourceBundleELResolver (Beans Binding)


---


|  |  |  |
| --- | --- | --- |
| **Overview** \| **Package** \| **Class** \| **Use** \| **Tree** \| **Deprecated** \| **Index** \| **Help** | |  |
| **PREV CLASS**   **NEXT CLASS** | **FRAMES**    **NO FRAMES**     **All Classes** |  |
| SUMMARY: NESTED \| FIELD \| CONSTR \| METHOD | DETAIL: FIELD \| CONSTR \| METHOD |  |


---


## org.jdesktop.el Class ResourceBundleELResolver

```
java.lang.Object
  org.jdesktop.el.ELResolver
      org.jdesktop.el.ResourceBundleELResolver
```

---

``` public class ResourceBundleELResolver extends ELResolver ```

Defines property resolution behavior on instances of
`ResourceBundle`.

This resolver handles base objects of type
`java.util.ResourceBundle`. It accepts any object as a property
and coerces it to a `java.lang.String` for invoking
`ResourceBundle.getObject(java.lang.String)`.

This resolver is read only and will throw a
`PropertyNotWritableException` if `setValue` is called.

`ELResolver`s are combined together using
`CompositeELResolver`s, to define rich semantics for evaluating an
expression. See the javadocs for `ELResolver` for details.

**Since:**
:   JSP 2.1

**See Also:**: `CompositeELResolver`, `ELResolver`, `ResourceBundle`

---

**Field Summary**

**Fields inherited from class org.jdesktop.el.ELResolver**

`RESOLVABLE_AT_DESIGN_TIME, TYPE`


**Constructor Summary**

`ResourceBundleELResolver()`


| **Method Summary** | |
| --- | --- |
| `java.lang.Class<?>` | `getCommonPropertyType(ELContext context, java.lang.Object base)`             If the base object is a ResourceBundle, returns the most general type that this resolver accepts for the `property` argument. |
| `java.util.Iterator` | `getFeatureDescriptors(ELContext context, java.lang.Object base)`             If the base object is a ResourceBundle, returns an `Iterator` containing the set of keys available in the `ResourceBundle`. |
| `java.lang.Class<?>` | `getType(ELContext context, java.lang.Object base, java.lang.Object property)`             If the base object is an instance of `ResourceBundle`, return `null`, since the resolver is read only. |
| `java.lang.Object` | `getValue(ELContext context, java.lang.Object base, java.lang.Object property)`             If the base object is an instance of `ResourceBundle`, the provided property will first be coerced to a `String`. |
| `boolean` | `isReadOnly(ELContext context, java.lang.Object base, java.lang.Object property)`             If the base object is not null and an instanceof `ResourceBundle`, return `true`. |
| `void` | `setValue(ELContext context, java.lang.Object base, java.lang.Object property, java.lang.Object value)`             If the base object is a ResourceBundle, throw a `PropertyNotWritableException`. |

**Methods inherited from class java.lang.Object**

`clone, equals, finalize, getClass, hashCode, notify, notifyAll, toString, wait, wait, wait`

**Constructor Detail**

### ResourceBundleELResolver

```
public ResourceBundleELResolver()
```


**Method Detail**

### getValue

```
public java.lang.Object getValue(ELContext context,
                                 java.lang.Object base,
                                 java.lang.Object property)
```

:   If the base object is an instance of `ResourceBundle`,
    the provided property will first be coerced to a `String`.
    The `Object` returned by `getObject` on
    the base `ResourceBundle` will be returned.

If the base is `ResourceBundle`, the
`propertyResolved` property of the `ELContext`
object must be set to `true` by this resolver, before
returning. If this property is not `true` after this method
is called, the caller should ignore the return value.

:   **Specified by:**: `getValue` in class `ELResolver`
:   **Parameters:**: `context` - The context of this evaluation.: `base` - The ResourceBundle to analyze.: `property` - The name of the property to analyze. Will be coerced to a `String`. **Returns:**: If the `propertyResolved` property of `ELContext` was set to `true`, then `null` if property is `null`; otherwise the `Object` for the given key (property coerced to `String`) from the `ResourceBundle`. If no object for the given key can be found, then the `String` "???" + key + "???". **Throws:**: `java.lang.NullPointerException` - if context is `null`: `ELException` - if an exception was thrown while performing the property or variable resolution. The thrown exception must be included as the cause property of this exception, if available.


---


### getType

```
public java.lang.Class<?> getType(ELContext context,
                                  java.lang.Object base,
                                  java.lang.Object property)
```

:   If the base object is an instance of `ResourceBundle`,
    return `null`, since the resolver is read only.

    If the base is `ResourceBundle`, the
    `propertyResolved` property of the `ELContext`
    object must be set to `true` by this resolver, before
    returning. If this property is not `true` after this method
    is called, the caller should ignore the return value.

    :   **Specified by:**: `getType` in class `ELResolver`
    :   **Parameters:**: `context` - The context of this evaluation.: `base` - The ResourceBundle to analyze.: `property` - The name of the property to analyze. **Returns:**: If the `propertyResolved` property of `ELContext` was set to `true`, then `null`; otherwise undefined. **Throws:**: `java.lang.NullPointerException` - if context is `null`

---


### setValue

```
public void setValue(ELContext context,
                     java.lang.Object base,
                     java.lang.Object property,
                     java.lang.Object value)
```

:   If the base object is a ResourceBundle, throw a
    `PropertyNotWritableException`.

    :   **Specified by:**: `setValue` in class `ELResolver`
    :   **Parameters:**: `context` - The context of this evaluation.: `base` - The ResourceBundle to be modified. Only bases that are of type ResourceBundle are handled.: `property` - The String property to use.: `value` - The value to be set. **Throws:**: `java.lang.NullPointerException` - if context is `null`.: `PropertyNotWritableException` - Always thrown if base is an instance of ReasourceBundle.

---


### isReadOnly

```
public boolean isReadOnly(ELContext context,
                          java.lang.Object base,
                          java.lang.Object property)
```

:   If the base object is not null and an instanceof `ResourceBundle`,
    return `true`.

    :   **Specified by:**: `isReadOnly` in class `ELResolver`
    :   **Parameters:**: `context` - The context of this evaluation.: `base` - The ResourceBundle to be modified. Only bases that are of type ResourceBundle are handled.: `property` - The String property to use. **Returns:**: If the `propertyResolved` property of `ELContext` was set to `true`, then `true`; otherwise undefined. **Throws:**: `java.lang.NullPointerException` - if context is `null`

---


### getFeatureDescriptors

```
public java.util.Iterator getFeatureDescriptors(ELContext context,
                                                java.lang.Object base)
```

:   If the base object is a ResourceBundle, returns an `Iterator`
    containing the set of keys available in the `ResourceBundle`.
    Otherwise, returns `null`.

    The `Iterator` returned must contain zero or more instances
    of `FeatureDescriptor`. Each info object contains
    information about a key in the ResourceBundle, and is initialized as
    follows:

    - displayName - The `String` key- name - Same as displayName property.
      - shortDescription - Empty string
      - expert - `false`
      - hidden - `false`
      - preferred - `true`

    In addition, the following named attributes must be set in the returned
    `FeatureDescriptor`s:

    - `ELResolver.TYPE` - `String.class`
    - `ELResolver.RESOLVABLE_AT_DESIGN_TIME` - `true`

    :   **Specified by:**: `getFeatureDescriptors` in class `ELResolver`
    :   **Parameters:**: `context` - The context of this evaluation.: `base` - The bundle whose keys are to be iterated over. Only bases of type `ResourceBundle` are handled by this resolver. **Returns:**: An `Iterator` containing zero or more (possibly infinitely more) `FeatureDescriptor` objects, each representing a key in this bundle, or `null` if the base object is not a ResourceBundle. **See Also:**: `FeatureDescriptor`

---


### getCommonPropertyType

```
public java.lang.Class<?> getCommonPropertyType(ELContext context,
                                                java.lang.Object base)
```

:   If the base object is a ResourceBundle, returns the most general type
    that this resolver accepts for the `property` argument.
    Otherwise, returns `null`.

    Assuming the base is a `ResourceBundle`, this method will
    always return `String.class`.

    :   **Specified by:**: `getCommonPropertyType` in class `ELResolver`
    :   **Parameters:**: `context` - The context of this evaluation.: `base` - The bundle to analyze. Only bases of type `ResourceBundle` are handled by this resolver. **Returns:**: `null` if base is not a `ResourceBundle`; otherwise `String.class`.


---


|  |  |  |
| --- | --- | --- |
| **Overview** \| **Package** \| **Class** \| **Use** \| **Tree** \| **Deprecated** \| **Index** \| **Help** | |  |
| **PREV CLASS**   **NEXT CLASS** | **FRAMES**    **NO FRAMES**     **All Classes** |  |
| SUMMARY: NESTED \| FIELD \| CONSTR \| METHOD | DETAIL: FIELD \| CONSTR \| METHOD |  |


---
